# A Unique Case of Odontoid Metastasis Reveals Nasopharynx Carcinoma: Case Management With the Posterolateral Epidural Supra-C2-Root Approach (PESCA) Technique in Open Surgery

**DOI:** 10.7759/cureus.117248

**Published:** 2026-09-30

**Authors:** Mehfoud Ibrahim, Mohamed Khoulali, Mohamed Dahamou, Nadir Miry, Faycal Moufid

**Affiliations:** 1 Department of Neurosurgery, Mohammed VI University Hospital of Oujda, Faculty of Medicine and Pharmacy of Oujda, Mohammed First University Oujda, Oujda, MAR; 2 Department of Histopathology, Mohammed VI University Hospital of Oujda, Faculty of Medicine and Pharmacy of Oujda, Mohammed First University Oujda, Oujda, MAR; 3 Department of Pathology, Mohammed VI University Hospital of Oujda, Faculty of Medicine and Pharmacy of Oujda, Mohammed First University Oujda, Oujda, MAR

**Keywords:** cavum, metastasis, odontoid, odontoid lesion, pesca, posterolateral epidural supra-c2-root approach (pesca)

## Abstract

We present a unique case of a 21-year-old boy diagnosed with an unstable odontoid due to cavum metastasis revealed by cervicalgia and hyperextension cervical spine deformity. There were three main problems in this case: odontoid instability, the correction of cervico-occipital deformity, and histological proof. The posterior approach with the posterolateral epidural supra-C2-root approach (PESCA) resolved the situation with great improvement. This challenging finding was unusual, and in such a situation, a large review of the literature was made to make the right choice for the patient to prevent major neurological deficits and even death.

## Introduction

The odontoid process, or dens, is a tooth-like peg sticking up from the C2 vertebra (the axis). On the front, it has a smooth oval spot where it connects with the front ring of C1 (the atlas), letting the neck turn. Around the back, a small groove cradles the transverse ligament, which locks the peg securely in place so it doesn't shift into the spinal cord. Right near the pointed top, tough alar and apical ligaments lash the bone directly to the base of the skull for extra stability.

Squamous cell carcinoma of the nasopharynx can be classified into several histological types. We particularly distinguish between keratinizing differentiated squamous cell carcinoma and non-keratinizing differentiated squamous cell carcinoma. It is closely linked to infection by the Epstein-Barr virus (EBV). These types present differences in epidemiology and histological characteristics, making them important to consider in the diagnosis and treatment of nasopharyngeal cancers.

The most frequent metastatic sites outside of local and cervical lymph node metastases are bony (70%), pulmonary, hepatic, and extracervical lymph nodes [1]. While bone metastases are common in this context, vertebral metastases, particularly at the odontoid level, are very occasional and rare. That is why we report here a unique case of a metastasis from a nasopharyngeal carcinoma at this level, with a management strategy using the posterolateral epidural supra-C2-root approach (PESCA) as well as a literature review.

## Case presentation

A 21-year-old male, with no notable pathological history, was admitted for the management of progressively worsening neck pain with neck extension. The patient showed no neurological signs or signs of spinal cord irritation. Furthermore, the patient has no ENT signs, including nasopharyngeal mass syndrome, dysphagia, or epistaxis.

Biological investigations, including a CBC, CRP, renal function tests, standard preoperative panel, and sputum tuberculosis research, showed no significant abnormalities. A standard X-ray showed a C1-C2 dislocation (Figure 1).

**Figure 1:**
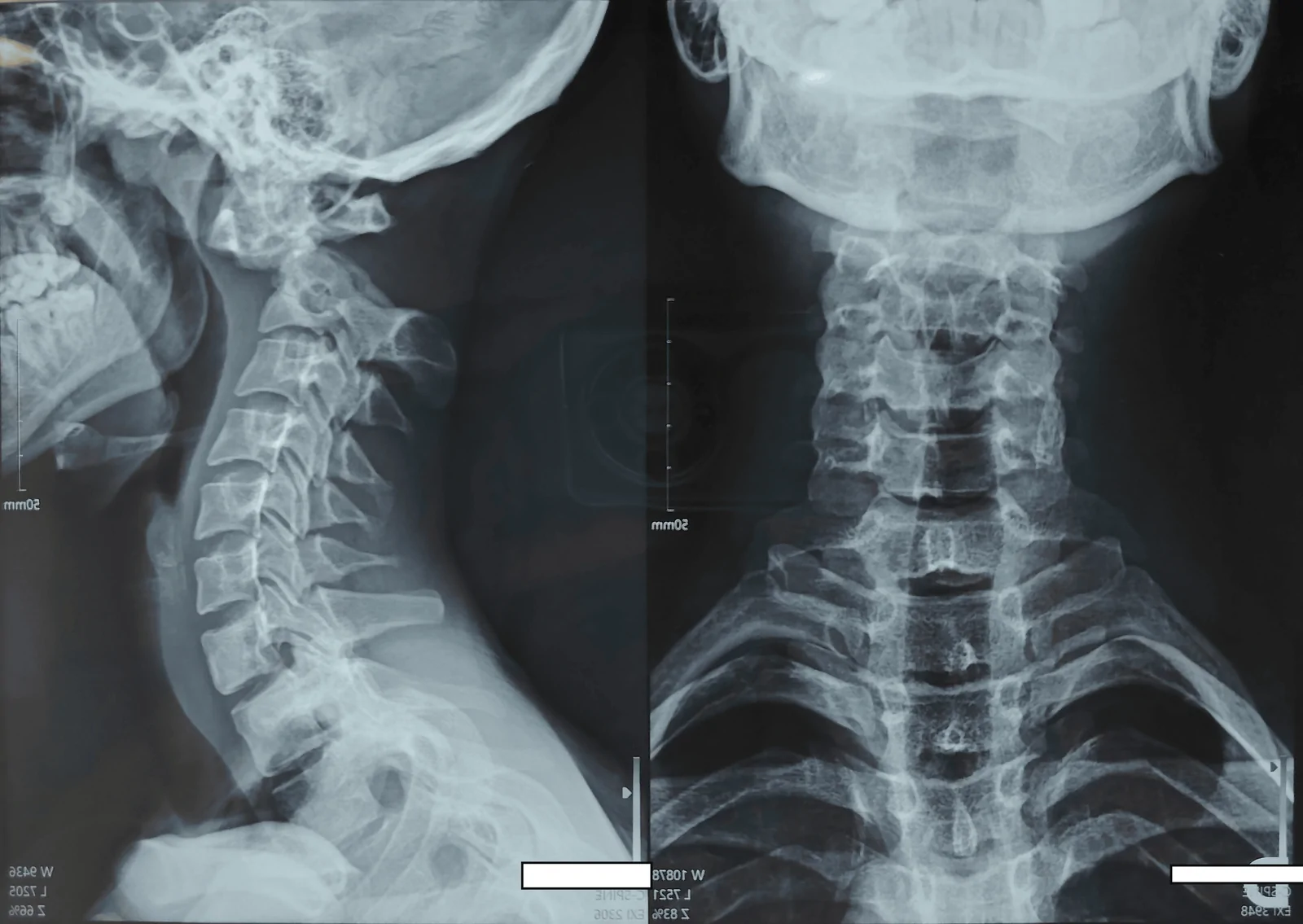
Anteroposterior (right panel) and lateral (left panel) plain radiographs of the cervical spine show the cervical vertebral alignment in a hyperextended position with C1-C2 disclocation.

A computed tomography arterial portography (CTAP) showed a lesion of the odontoid process of C2, a vertebral body lesion of C7 and L5, and a thinning of the posterolateral walls of the cavity with suspicious laterocervical adenopathy (Figure 2).

**Figure 2:**
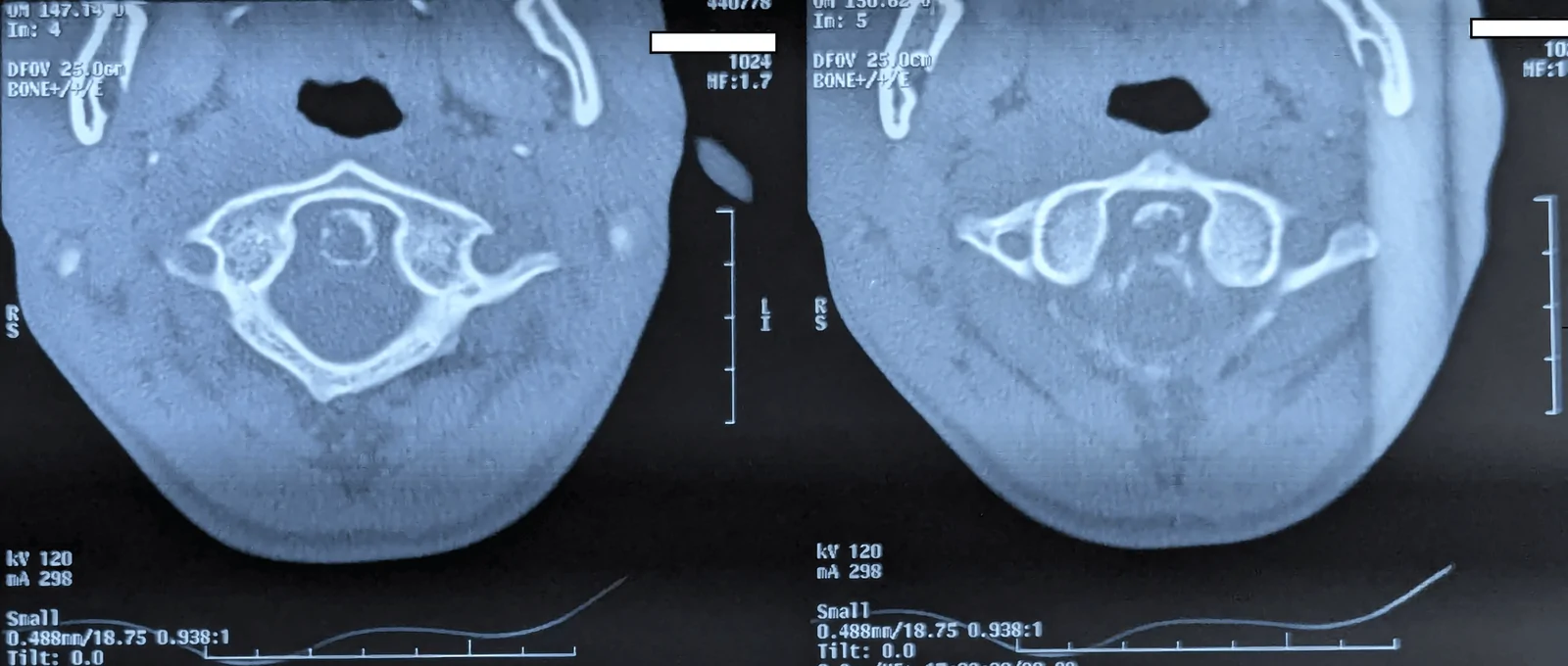
The axial CT images at the level of C2 reveal an abnormal lesion involving the odontoid process (dens). The cortical margins are disrupted with irregularity and heterogeneous attenuation. There is clear lytic involvement of the dens, associated with cortical thinning and partial osseous destruction.

Cervical MRI confirmed these lesions with instability of the C1-C2 joint and showed a nasopharyngeal tumoral lesion (Figure 3). Given the C1-C2 instability, a neurosurgical indication was retained with biopsy of the C2 dent.

**Figure 3:**
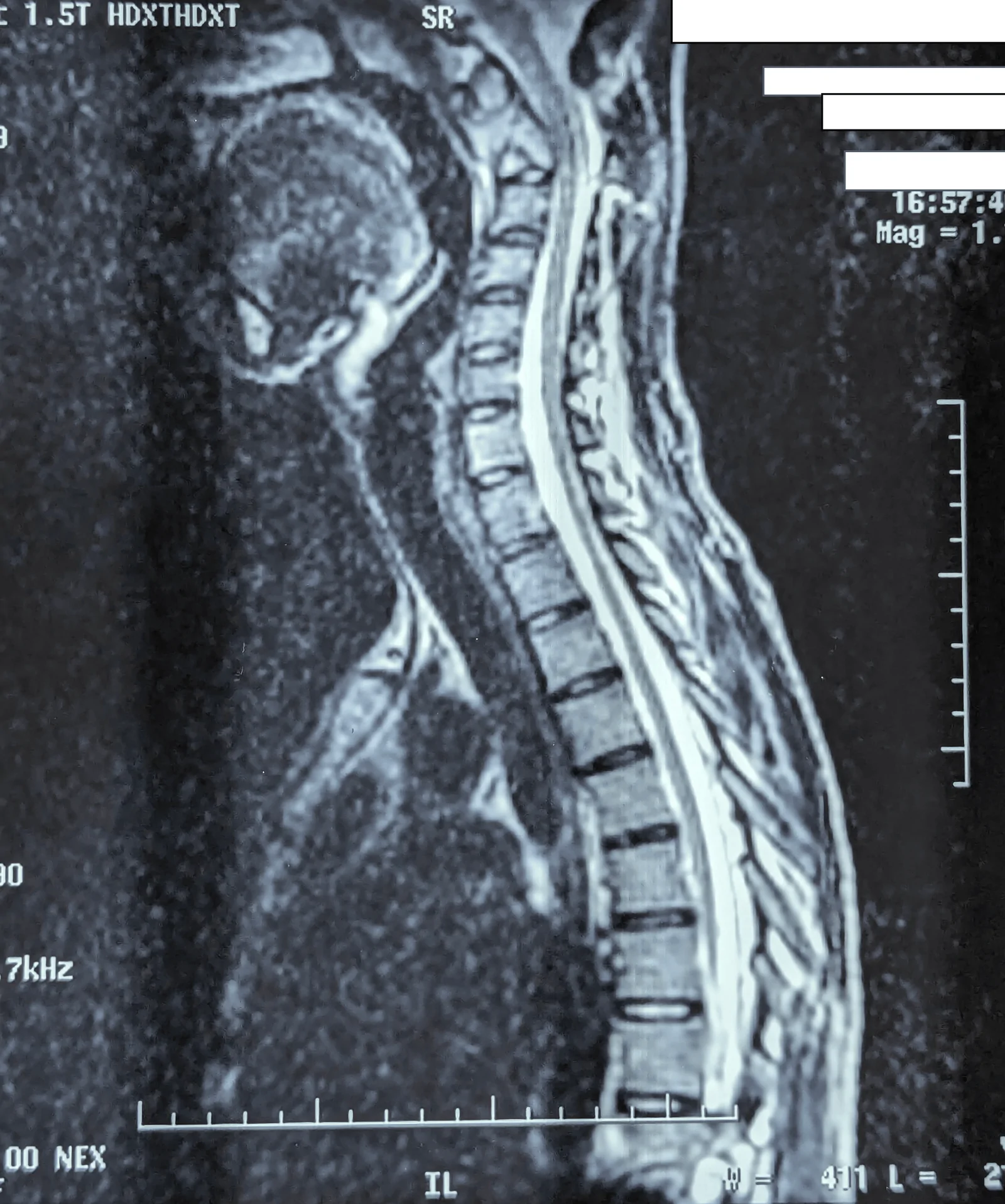
Sagittal T2-weighted MRI of the cervical spine demonstrates a destructive lesion of the odontoid process (C2 dens) with cortical disruption and heterogeneous signal intensity, consistent with a pathological process. There is an anterior thecal sac indentation without significant cord compression.

The patient was placed in a prone position with the head secured in a Mayfield clamp and under fluoroscopy, and a midline incision was made from the occiput to the target cervical level, usually C0 or C4 (Figure 4).

**Figure 4:**
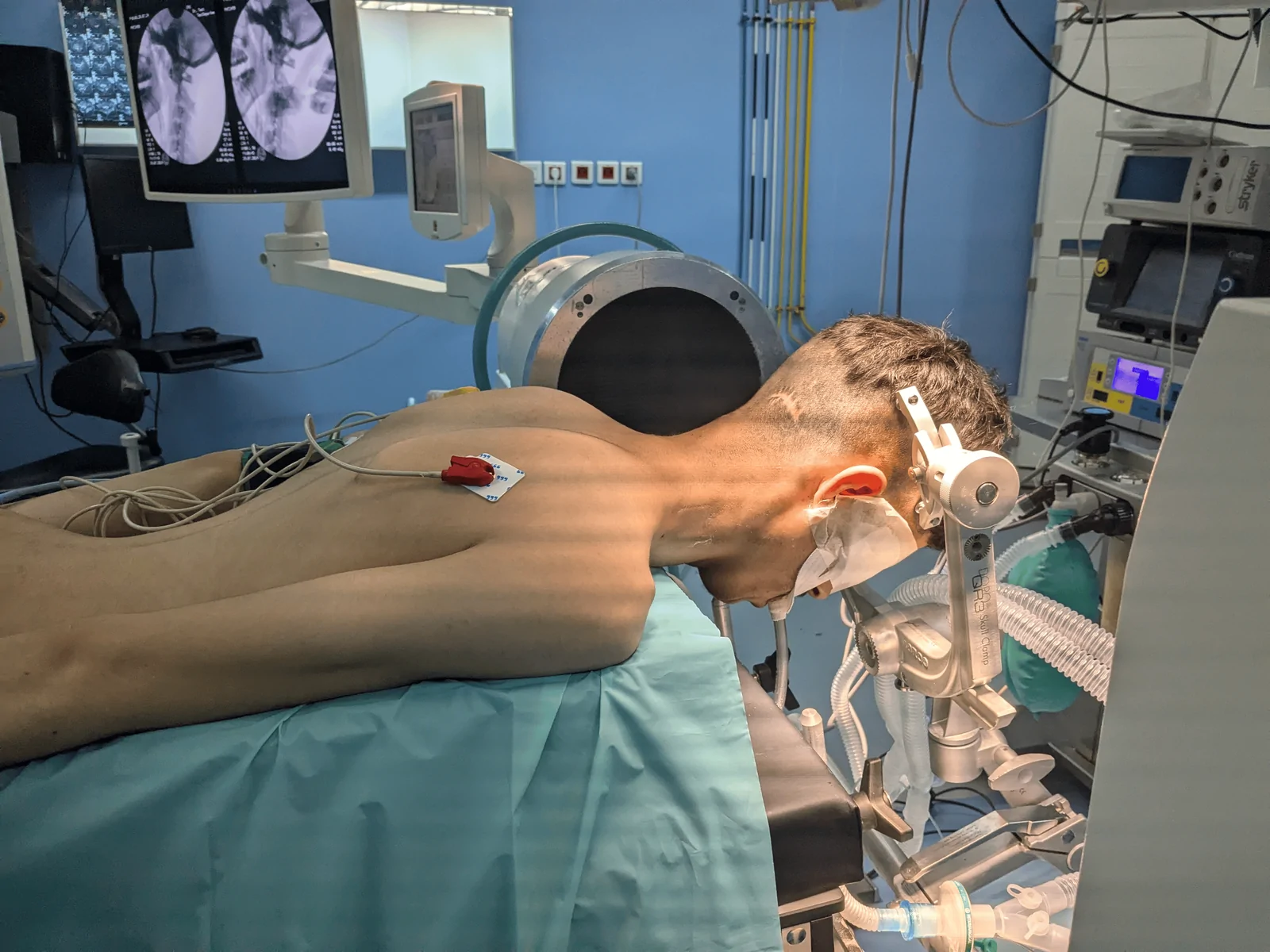
The preoperative picture shows the patient in a ventral position with the head in a normal position fixed with a Mayfield clamp.

Then, an incision was made from C0 to C4. The Vertex Select Reconstruction System (Medtronic, Memphis, TN, USA) from occipital C0 to C3-C4 was used for fixation. By diamond burr microdrilling, a laminectomy of the inferior part of the C1 arch was performed, and then it was completed by the removal of the C1 lateral mass and the superior part of the C2 arch. This created a tiny functional corridor directly above the C2 nerve root, through an epidural path, lateral-medullary route; a small biopsy from the dent was successfully obtained. The biopsy obtained was tan-grey and fibro-elastic in consistency (Figure 5).

**Figure 5:**
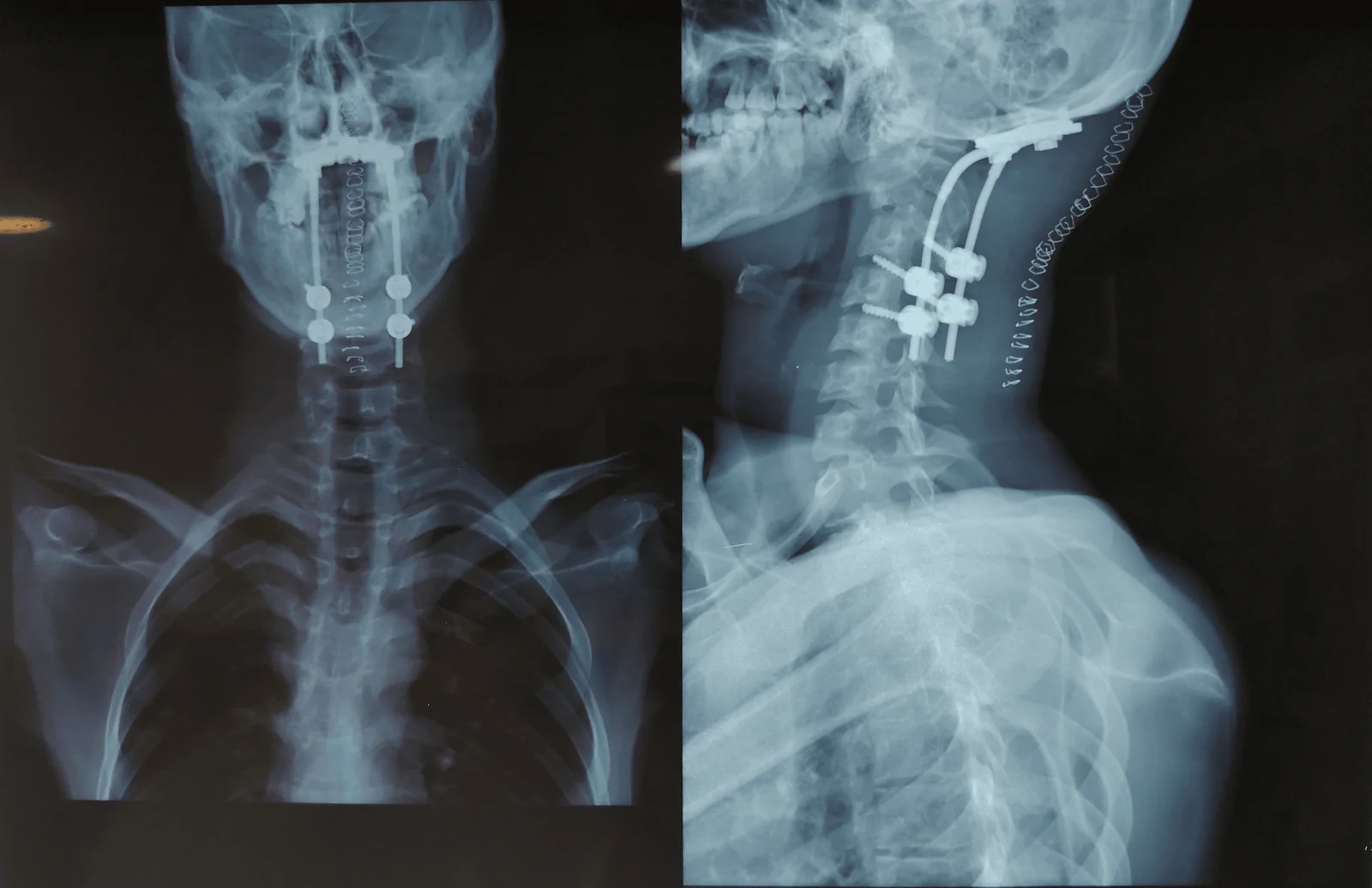
Postoperative anteroposterior (right panel) and lateral (left panel) plain radiographs of the cervical spine show posterior cervical fixation with the Vertex Select Reconstruction System (Medtronic, Memphis, TN, USA) and laminectomy and restoration of head position.

Histopathological and immunohistochemical examination showed that photomicrographs of the lesion revealed a proliferation arranged in solid sheets, composed of atypical cells with enlarged nuclei and abundant eosinophilic cytoplasm (Figures 6, 7). Immunohistochemical staining demonstrated nuclear positivity for P63 (Figure 8).

**Figure 6:**
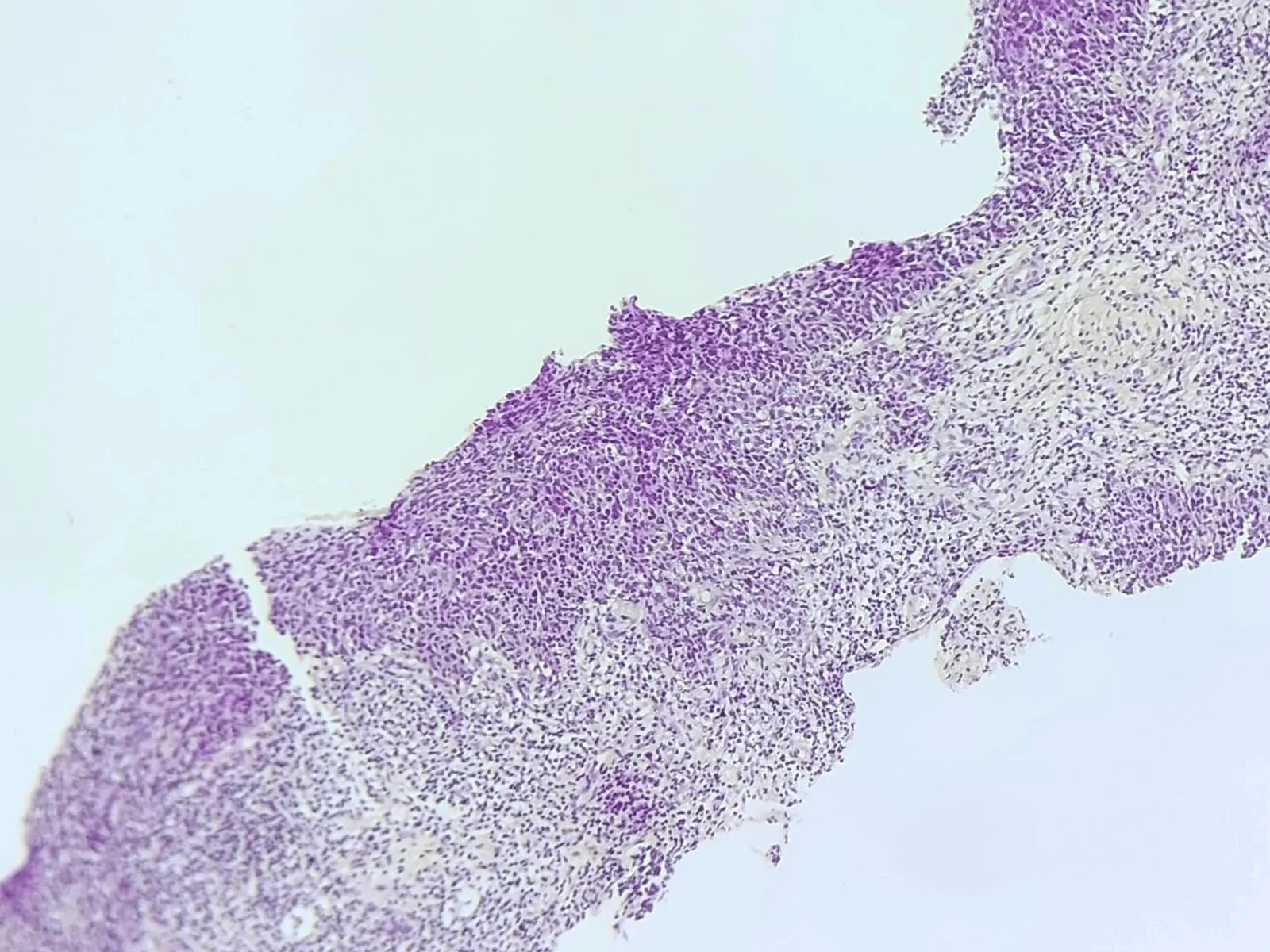
Photomicrographs of the lesion reveal a proliferation arranged in solid sheets, composed of atypical cells with enlarged nuclei and abundant eosinophilic cytoplasm.

**Figure 7:**
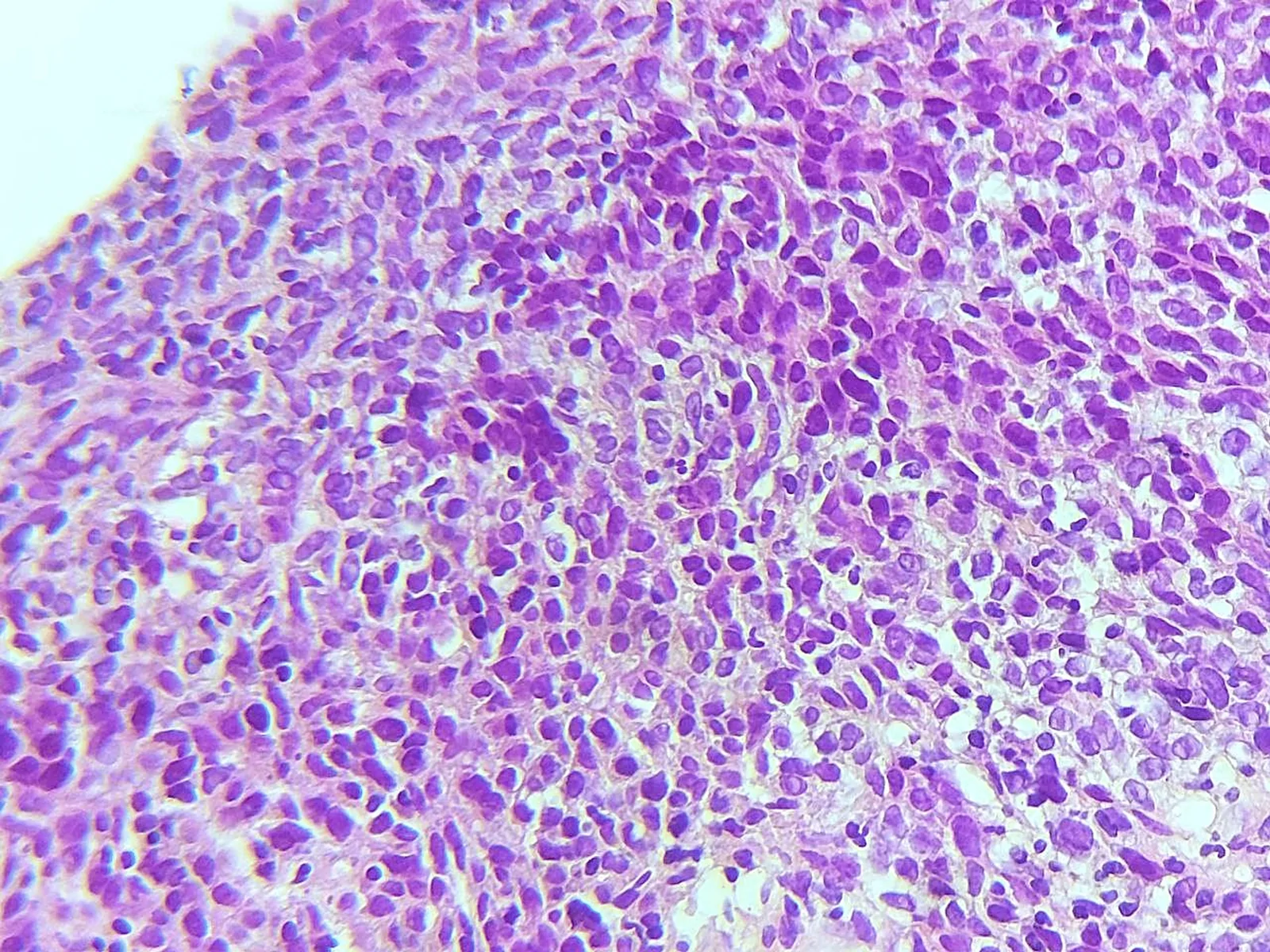
Photomicrographs of the lesion reveal a proliferation arranged in solid sheets, composed of atypical cells with enlarged nuclei and abundant eosinophilic cytoplasm.

**Figure 8:**
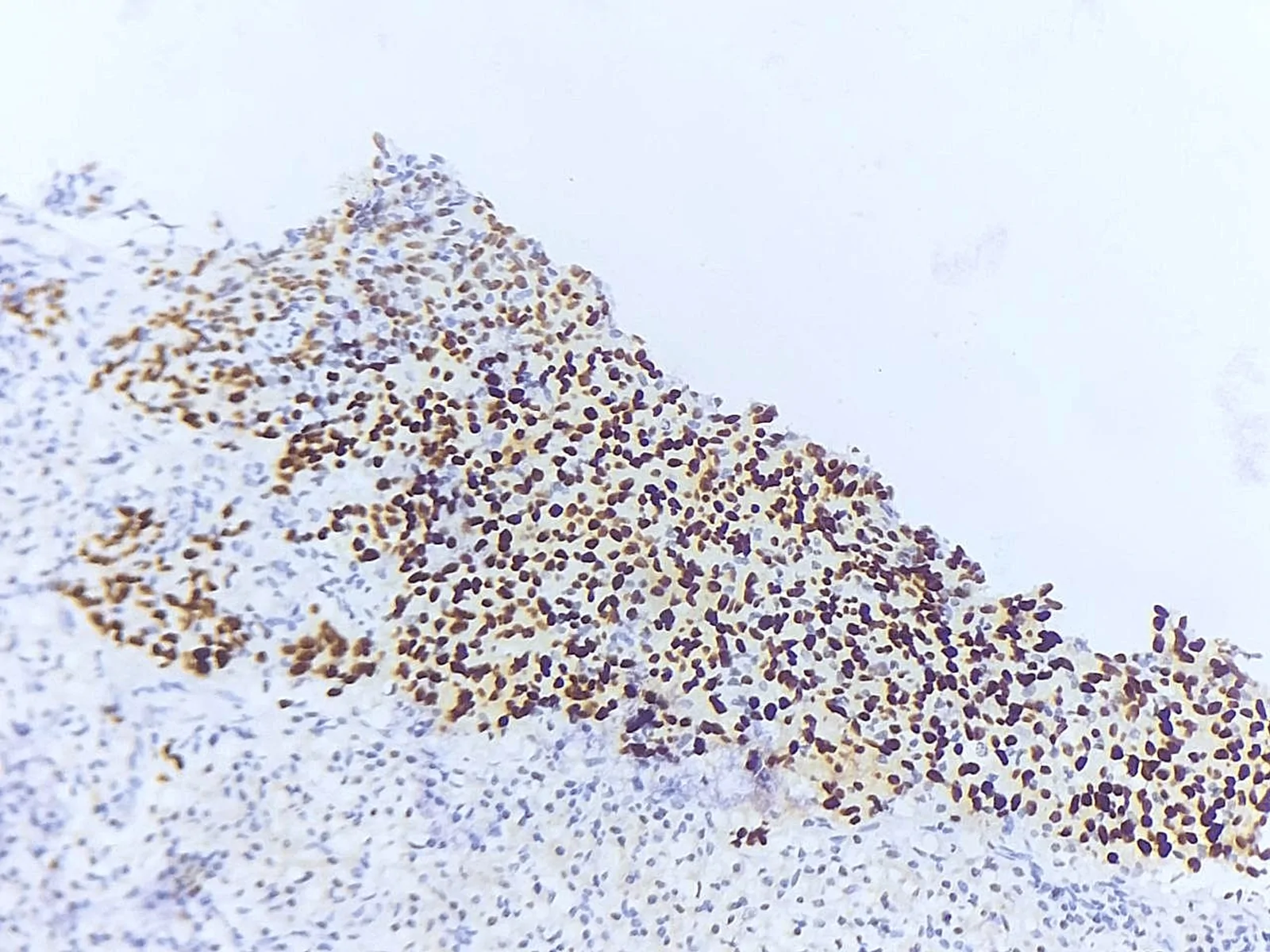
Immunohistochemical staining demonstrates nuclear positivity for P63.

The patient made a remarkable recovery. The postoperative course was uneventful, and the patient was discharged home three days after surgery without any complications. At the three-month follow-up, the patient reported remarkable amelioration of the cervical attitude and appearance of dysphagia. We referred the patient to ENT for evaluation and management of dysphagia, as well as ongoing oncological follow-up. He is currently undergoing radiochemotherapy.

## Discussion

Spinal metastases are a common manifestation of systemic cancer, representing the most frequent type of spinal tumor. They occur when malignant cells from a primary tumor spread hematogenously or, less commonly, via lymphatic or direct extension to the vertebral column [2]. The thoracic spine is most frequently affected, followed by the lumbar and cervical regions. Clinically, spinal metastases may present with axial pain, mechanical instability, radiculopathy, or myelopathy due to spinal cord or nerve root compression [3]. The goals of management include pain control, preservation or restoration of neurological function, and maintenance of spinal stability, often requiring a multidisciplinary approach involving oncology, radiology, and spine surgery [3-5].

Metastatic involvement of the cervical spine, while less common than in other regions, accounts for less than 10% [3,4]. It carries particular risks due to its proximity to vital neural and vascular structures. The atlantoaxial region (C1-C2) is an unusual site for metastases, accounting for a small fraction of cervical cases in 2021 (fewer than 15 cases) [2].

This high cervical location is biomechanically critical for head rotation and stability, and tumor invasion here can rapidly lead to instability, severe pain, and neurological compromise. Primary tumors that most commonly metastasize to the cervical spine include breast, lung, prostate, renal, and thyroid carcinomas [3-4]. Diagnosis typically relies on MRI for soft tissue and neural assessment, complemented by CT to evaluate bony destruction and stability [2-5]. 

The odontoid process (dens) of the axis (C2) is a rare but clinically significant site for metastatic disease. Because of its unique anatomical role in craniocervical stability and motion, lesions affecting the dens can cause severe mechanical instability and a high risk of spinal cord compression at the cervicomedullary junction [2-6]. Symptoms often include persistent occipital or suboccipital pain exacerbated by head movement, and in advanced cases, myelopathic signs such as weakness, hyperreflexia, or respiratory compromise [3]. Given the high functional importance of the dens, even small lesions may result in disproportionate clinical impact compared to metastases in other spinal locations.

Management of metastatic lesions to the odontoid requires careful balancing of oncologic control, neurological preservation, and mechanical stabilization. Non-surgical measures such as radiotherapy (including stereotactic radiosurgery) and systemic therapy may be effective in radiosensitive tumors without gross instability [3]. However, in cases of structural compromise, surgical stabilization, often via posterior occipitocervical fusion or transoral/transnasal approaches for tumor resection, may be indicated [7,8]. Prognosis depends largely on the primary tumor type, extent of systemic disease, and the patient’s overall functional status, but early recognition and intervention are critical to prevent catastrophic neurological deterioration.

Nasopharyngeal carcinoma (NPC) is characterized by early and widespread lymphatic dissemination, with the majority of patients exhibiting cervical lymph node involvement, as well as distant hematogenous metastases to the bone, lung, and liver [9,10].

Clinically significant upper-cervical (C1-C2) involvement, and especially odontoid lesions, are uncommon despite spinal metastases from NPC. When they do occur, they endanger atlantoaxial stability and cord integrity, necessitating immediate stabilization and palliative control (surgery, radiosurgery, and systemic therapy) [10,11].

One major risk is misinterpreting radiation-induced osteoradionecrosis (ORN) of C1-C2 as metastases because many NPC survivors have received radiation therapy: On MRI/PET, ORN can resemble lytic densopathy and even result in basilar invagination or atlantoaxial subluxation [11].

Because management varies (antibiotics/hyperbaric oxygen therapy/debridement vs. radiotherapy/systemic therapy), the work-up of a suspected dens lesion in NPC should, therefore, incorporate MRI (with attention to marrow signal, cortical breach, and adjacent soft-tissue), PET-CT, and plasma EBV-DNA, and when possible tissue confirmation to distinguish ORN from metastasis [12-14].

Neurosurgical goals for unstable C2 disease (destructive ORN or metastases) are pain management, decompression where necessary, and long-term stabilization [12-15].

For our patient, we used the Vertex Select Reconstruction System to stabilize the occipitocervical junction, which is the perfect choice for this kind of situation due to odontoid destruction. The Vertex Select Reconstruction System is a biomechanically strong and versatile fixation system that offers reliable fixation, reduces soft tissue irritation, and is compatible with standard cervical fixation systems, enhancing fusion rates and facilitating bone graft incorporation and minimizing micromotion [16,17].

The surgical technique follows a standard posterior midline approach. The patient is placed in a prone position with the head secured in a Mayfield clamp, and a midline incision is made from the occiput to the target cervical level, usually C0 or C4. After subperiosteal dissection, the occipital squama and posterior cervical elements are exposed. The occipital plate is contoured to fit the skull, and bicortical screws are placed in the midline keel, which offers the thickest and strongest bone purchase. Cervical fixation is then achieved by inserting screws into the translaminar of C3-C4. Pre-bent rods are then connected between the occipital plate and the cervical screws using the Vertex modular connectors. Proper alignment and reduction are verified with fluoroscopy; it is followed by autograft placement with the spinous process to promote fusion [18,19].

PESCA provides a convenient and secure method for biopsies of odontoid process lesions. The midsection of the C1 arch and a segment of the lateral C1 arc are removed once the craniocervical junction has been stabilized. This creates a tiny functional corridor directly above the C2 nerve root. In our case, this was obtained by laminectomy of the C1 arch, and then we just completed it by the removal of the C1 lateral mass [20].

This corridor is naturally protected by the spinal cord medially, the vertebral artery laterally, the C2 root, and the dura below. This makes it possible to guide a biopsy tool anteromedially toward the dens or retro-odontoid mass. Compared to transoral or transdural methods, this technique has a lower risk of infection, prevents tugging on the spinal cord, and keeps the vertebral artery protected by bone [20,21].

## Conclusions

Posterior access to the odontoid process presents a significant challenge for many neurosurgeons, especially when both diagnostic biopsy and structural stabilization are necessary. The PESCA technique provides a trustworthy anatomical corridor for safe tissue. Although immediate postoperative outcomes were favorable and no intraoperative complications occurred, long-term monitoring seems obligatory. The occurrence of dysphagia underscores the need to distinguish between surgical sequelae and disease progression to ensure comprehensive oncologic management.

## References

[1] Bensouda Y, Kaikani W, Ahbeddou N (2011). Treatment for metastatic nasopharyngeal carcinoma. Eur Ann Otorhinolaryngol Head Neck Dis.

[2] Pongmanee S, Sarasombath P, Rojdumrongrattana B, Liawrungrueang W (2022). An unusual chordoma of the odontoid process: a case report and literature review. J Am Acad Orthop Surg Glob Res Rev.

[3] Saad A, Azzopardi C, Haleem S, Czyz M, James SL, Botchu R (2020). Tumours of the odontoid peg revisited. Indian J Radiol Imaging.

[4] Lakemeier S, Westhoff CC, Fuchs-Winkelmann S, Schofer MD (2009). Odontoid process metastasis of bronchial carcinoma as a rare cause for nonmechanical neck pain: a case report. Cases J.

[5] Rayan F, Mukundan C, Shukla DD, Barrington RL (2009). Odontoid metastasis: a potential lethal complication. J Orthop Traumatol.

[6] Gaillard F, Bell D, Jones J (2026). Erosion of the odontoid process (differential). https://radiopaedia.org/articles/erosion-of-the-odontoid-process-differential?lang=us.

[7] Voelker A, von der Hoeh NH, Heyde CE (2018). Balloon kyphoplasty and additional anterior odontoid screw fixation for treatment of unstable osteolytic lesions of the vertebral body C2: a case series. BMC Musculoskelet Disord.

[8] Papp Z, Marosfői M, Szikora I, Banczerowski P (2014). Treatment of C-2 metastatic tumors with intraoperative transoral or transpedicular vertebroplasty and occipitocervical posterior fixation. J Neurosurg Spine.

[9] Yang J, Hu J, Wang D, Jia Q, Jiao J, Xiao J (2020). Surgical treatment outcomes of spinal metastases of nasopharyngeal carcinoma: the first report of 30 patients from a single center. Cancer Manag Res.

[10] Ho FC, Tham IW, Earnest A, Lee KM, Lu JJ (2012). Patterns of regional lymph node metastasis of nasopharyngeal carcinoma: a meta-analysis of clinical evidence. BMC Cancer.

[11] Lee JS, Huang CM, Yeh IC, Chen HH, Lin JC, Chen HH (2010). Isolated osteoradionecrosis of the dens mimicking metastasis of nasopharyngeal carcinoma after radiotherapy. J Clin Neurosci.

[12] Yang J, Jia Q, Peng D (2017). Surgical treatment of upper cervical spine metastases: a retrospective study of 39 cases. World J Surg Oncol.

[13] Zhong X, Li L, Lu B (2020). Differentiation of cervical spine osteoradionecrosis and bone metastasis after radiotherapy detected by bone scan in patients with nasopharyngeal carcinoma: role of magnetic resonance imaging. Front Oncol.

[14] Kim KM, Yoon JH, Kwon JW, Gwak HS (2019). Transoral vertebroplasty for osteolytic C2 metastasis: report of 2 cases. Nerve.

[15] Chen Z, Bi Z, Liu D (2024). Endoscopic transnasal and transoral resection of the odontoid process and C1 combined with occipitocervical fusion for osteoradionecrosis of the upper cervical spine: a case report and literature review. BMC Neurol.

[16] VERTEX SELECT (2026). Vertex Select. https://neurospineproductreview.com/storage/products/pdfs/st-vertex-selectr-c1-c7-screw-fixation-surgical-technique.pdf.

[17] Vertex Select Reconstruction System Indications (2026). Vertex Select Reconstruction System: indications, safety, and warnings. https://www.medtronic.com/en-ie/healthcare-professionals/products.html.

[18] Joaquim AF, Osorio JA, Riew KD (2020). Occipitocervical fixation: general considerations and surgical technique. Global Spine J.

[19] (2026). Cervical spine - posterior fixation. https://surgeryreference.aofoundation.org/spine/trauma/subaxial-cervical/c/cervical-spine-posterior-fixation.

[20] Haas P, Hauser TK, Kandilaris K, Skardelly M, Tatagiba M, Adib SD (2022). Case report: Posterolateral epidural supra-C2-root approach (PESCA) for biopsy of a retro-odontoid lesions in same sitting after occipitocervical fixation and decompression in a case of crowned dens syndrome with brainstem compression and displacement. Front Surg.

[21] Eissa EM, Eldin MM (2017). Odontoidectomy through posterior midline approach followed by same sitting occipitocervical fixation: a cadaveric study. J Craniovertebr Junction Spine.

